# Triptonide Suppresses AML via PI3K/AKT Signaling: A Network Pharmacology Approach Validated by Molecular Docking and Experimental Studies

**DOI:** 10.3390/cimb48030239

**Published:** 2026-02-24

**Authors:** Lixia Song, Jing Meng, Huijie Li, Wanxin Fu, Kun Hong, Shengnan Shen, Zheping Zhang, Shilan Ding, Shengpeng Li, Zifan Zhang, Weijian Bei, Hairu Huo, Yuqing Tan, Feng Sui, Li Liu

**Affiliations:** 1 Institute of Chinese Materia Medica, China Academy of Chinese Medical Sciences, Beijing 100700, China; 2 School of Life Sciences, Beijing University of Chinese Medicine, Beijing 102401, China; 3 State Key Laboratory for Quality Ensurance and Sustainable Use of Dao-di Herbs, Artemisinin Research Center, China Academy of Chinese Medical Sciences, Beijing 100700, China; 4 Institute of Chinese Medicinal Sciences, Guangdong TCM Key Laboratory against Metabolic Diseases, Guangdong Pharmaceutical University, Guangzhou 510006, China

**Keywords:** acute myeloid leukemia, triptonide, network pharmacology, molecular docking, molecular dynamics simulations

## Abstract

Triptonide (TN), a natural bioactive compound derived from *Tripterygium wilfordii* with multiple antitumor activities, has a poorly defined exact mechanism in acute myeloid leukemia (AML)—a hematologic malignancy with limited treatment options. This study systematically clarifies TN’s mechanisms in AML through an integrative strategy combining network pharmacology, molecular docking, molecular dynamics simulation, and in vitro/in vivo experiment validation. Predicted TN targets using Swiss Target Prediction and PharmMapper, and AML-associated genes via GeneCards, OMIM, and CTD. Verall, O198 overlapping targets were mapped to build a PPI network using STRING and Cytoscape. Identified hub gene (AKT1, EGFR, HSP90AA1, HSP90AB1, and PIK3R1) using CytoNCA, MCODE, and CytoHubba algorithms. GO and KEGG enrichment analyses highlighted marked enrichment in the PI3K/AKT pathway. TN exhibited high affinity binding to AKT1 (−7.28 kcal/mol) and PIK3R1 (−7.33 kcal/mol), with stable interactions confirmed by molecular dynamics simulations. The GSEA of the DEGs from GEPIA 2 revealed prominent activation of the PI3K/AKT signaling pathway, indicating its key role as a regulator of AML pathogenesis. In vitro, TN dose-dependently suppressed proliferation of multiple AML cell lines induced apoptosis, and downregulated the expression of P-PI3K and P-AKT. The AKT activator SC79 reversed TN-induced suppression in AML cells, validating PI3K/AKT pathway dependency. In vivo, TN significantly inhibited tumor growth in xenograft models without causing organ toxicity in female nude mice. These findings reveal the therapeutic potential of TN against AML through inhibiting the PI3K/AKT axis. With no PI3K/AKT inhibitors targeting AML approved as first-line therapies, TN emerges as a promising candidate for AML treatment, offering a safer natural alternative.

## 1. Introduction

Leukemia constitutes a malignant clonal neoplasm driven by abnormal proliferation of hematopoietic stem cells, characterized by uncontrolled self-renewal and differentiation defects, and is associated with elevated incidence rates, high mortality, and unfavorable survival rates. Epidemiological data from the American Cancer Society (2024) revealed that leukemia ranks 14th in cancer incidence and 6th/8th in mortality among males/females [1].

Acute myeloid leukemia (AML) represents a clonal malignancy with genetic diversity and is a major subtype of adult acute leukemia, comprising approximately 33% of new leukemia diagnoses [2,3]. Its incidence rates are positively correlated with advancing age [4]. The global burden of AML is experiencing significant transformation, driven by advances in medical technologies, population aging, and social factors [5,6,7].

The current therapeutic approaches for leukemia include chemotherapy, targeted therapy, and hematopoietic stem cell transplantation (HSCT) [8]. For newly diagnosed AML patients, the conventional first-line regimen employs the “3 + 7 protocol,” a combination of 3 days of anthracycline-based chemotherapy and 7 days of cytarabine, which achieves high complete remission rates but is associated with significant toxic side effects (e.g., myelosuppression, infection risk) and emerging drug resistance. For relapsed/refractory (R/R) or high-risk AML patients, allo-HSCT represents a potentially effective option. However, its clinical adoption is hindered by challenges, including donor availability, transplant-related mortality, and complications [8,9].

Recent advancements in targeted therapies and immunotherapies have demonstrated promising clinical potential. Despite advances in therapy, the 5-year overall survival of elderly (≥60 years) and R/R AML patients is persistently low (<20%) [10,11,12,13,14]. Therefore, there is an urgent demand for novel therapeutic approaches to minimize adverse effects and enhance clinical efficacy.

With the advancement of modern research on traditional Chinese medicine (TCM), herbal compounds have emerged as promising candidates for antitumor drug development because of their multitarget and multi-pathway mechanisms of action. Among these compounds, triptonide (TN), a bioactive diterpenoid lactone extracted from *Tripterygium wilfordii*, has garnered significant attention. Owing to its molecular formula C_20_H_22_O_6_ and molecular weight of 358.39 kDa, TN has broad biological activities, including immunomodulation [15], anti-inflammatory [16], insecticidal [17], and anti-fertility effects [18]. Emerging evidence highlights its potent anticancer activities in conditions such as pancreatic cancer [19], lymphoma [20], prostate cancer [20], gastric cancer [21,22], and lung cancer [23]. Recent studies have reported that TN exerts its antileukemic effect through the suppression of GLI2 and FLT3-ITD mutant proteins, thereby inhibiting cell proliferation and promoting apoptosis [24]; additionally, TN selectively activates the p38 MAPK signaling pathway, upregulating p16 and p21 to drive AML cell senescence while concurrently activating caspase-3/7 to promote apoptosis [15]. Notably, TN has superior safety profiles compared with conventional chemotherapeutic agents [18,25]. Our previous study preliminarily confirmed that TN significantly inhibits AML [26]. On the basis of these findings, the present study aimed to elucidate the molecular mechanisms underlying TN-mediated AML suppression and to systematically evaluate the treatment effect on AML.

**Figure 1:**
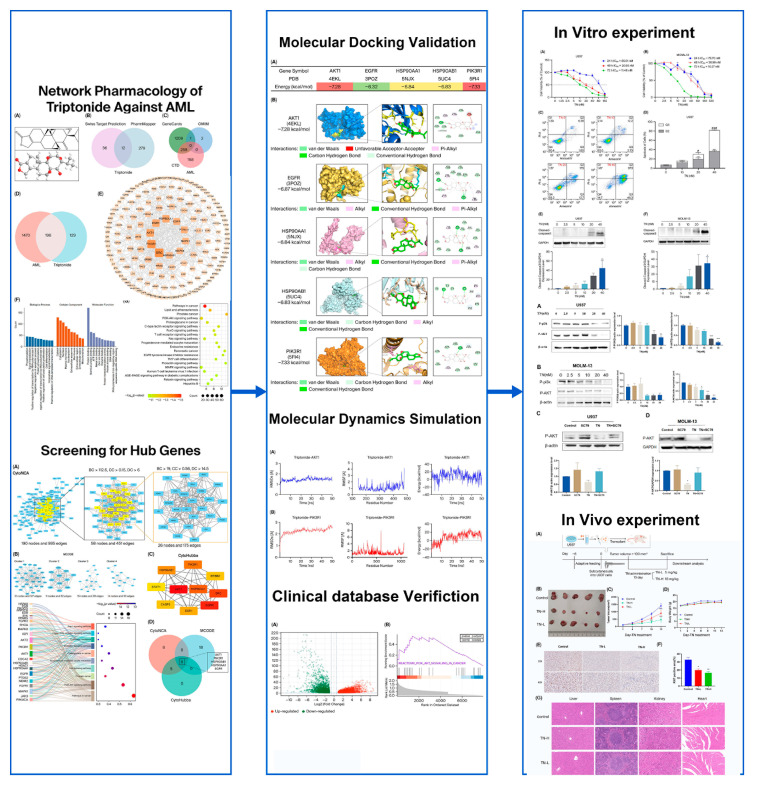
The flowchart reflecting our study design. Compared with the Control group (Q2), ** *p* < 0.01, *** *p* < 0.001; Compared with the Control group (Q3), ^#^ *p* < 0.05, ^###^ *p* < 0.001. Compare with the Control group, * *p* < 0.05, ** *p* < 0.01, *** *p* < 0.001.

In this research, we explored the mechanism of TN against AML by employing network pharmacology, molecular docking, molecular dynamics (MD) simulation, and experimental verification in vitro and in vivo. The flow diagram of the present study is presented in Figure 1.

## 2. Materials and Methods

### 2.1. Network Pharmacology Investigation of TN Against AML

#### 2.1.1. Target Prediction for TN

The potential targets of TN were revealed via searches of the SwissTargetPrediction database with “triptonide” as the keyword. The structure of TN was acquired from PubChem and subsequently submitted to PharmMapper for target identification. The targets predicted by both platforms were consolidated, and duplicates were removed for subsequent analysis. The URLs used in this section are listed in Table 1.

#### 2.1.2. Retrieval of AML-Associated Genes

Genes linked to AML were identified via the CTD, GeneCards, and OMIM databases with the keywords “acute myeloid leukemia” and the species “*Homo sapiens*”. We eliminated duplicates and created a comprehensive collection of relevant targets. The URLs used in this section are listed in Table 1.

#### 2.1.3. Identification of Common Targets and Construction of the PPI Network

The overlapping targets between AML and TN were visualized via a Venn diagram. These common targets were then submitted to the STRING database (version 12.0) to build a PPI network (minimum confidence score: 0.9), and disconnected nodes were hidden. The network was visualized and analyzed in Cytoscape (version 3.8.0).

#### 2.1.4. Functional Enrichment Analysis of the Common Targets

The overlapping targets were subjected to Gene Ontology (GO) and Kyoto Encyclopedia of Genes and Genomes (KEGG) enrichment analysis with the DAVID database. GO output was employed to generate a bar chart showing genes across molecular function (MF), cellular component (CC), and biological process (BP), while KEGG output was presented as a bubble diagram.

#### 2.1.5. Selection of Core Genes

To identify core genes, we integrated three Cytoscape (version 3.8.0) plugins—MCODE, CytoNCA, and CytoHubba—each capturing distinct topological features of the PPI network.

MCODE finds clusters (highly interconnected regions) within the network. Clusters mean different things in different types of networks. CytoHubba employs topological algorithms to predict and explore important nodes and subnetworks in a given network, ranked in the top 10 by maximal clique centrality (MCC) score were prioritized as candidate hubs. CytoNCA focuses on quantifying node centrality through classical metrics, including Degree Centrality (DC), Betweenness Centrality (BC), and Closeness Centrality (CC). Core targets were screened based on the median threshold value. This multi-method approach balanced local and global topological perspectives.

Key targets were determined by combining the results of the MCODE, CytoNCA, and CytoHubba algorithms. The hub targets were chosen on the basis of the degree of their respective medians.

### 2.2. Molecular Docking

We downloaded the structure of the TN from the PubChem database. The crystal structures of AKT1 (PDB: 4EKL), EGFR (PDB: 3POZ), HSP90AA1 (PDB: 5NJX), HSP90AB1 (PDB: 5UC4), and PIK3R1 (PDB: 5FI4) were retrieved from the PDB. Molecular docking was performed with AutoDock Vina 1.5.7, and protein-ligand optimization was performed with BIOVIA Discovery Studio (version 19.1.0.18287) (Dassault Systemes, Paris, France)and PyMOL (version 4.6.0) (Schrödinger, New York, NY, USA).

### 2.3. Molecular Dynamics Simulation

To evaluate the conformational dynamics and stability of the protein-ligand complexes, molecular dynamics (MD) simulations were employed using YASARA software (version 16.3.5) (YASARA Biosciences GmbH, Vienna, Austria) with the AMBER 14 force field. The receptor and ligand structures were preprocessed by removing solvent molecules, adding hydrogen atoms, and the assignment of protonation states (pH = 7.4) to optimize the structural integrity. A cubic simulation cell with a 5 Å extension around all atoms to enable periodic boundary conditions. Energy minimization was performed via steepest descent to relieve steric clashes and stabilize atomic interactions. Water and ions were added according to the software’s default settings. After the system was subjected to equilibration, a 50 ns (50,000 ps) MD simulation was performed, with real-time recording of parameters such as the root mean square fluctuations (RMSFs), root mean square deviations (RMSDs), and energy. Post-simulation, the resulting data were visualized using GraphPad Prism (version 8.0) (GraphPad Software, San Diego, CA, USA), with line graphs generated to facilitate intuitive interpretation of the analysis outcomes.

### 2.4. Data Collection and Processing

We obtained differentially expressed genes (DEGs) from the GEPIA 2 database by screening Normal samples and AML samples, with conditions of *p* < 0.05 and |log2FC| > 1. The DEGs were analyzed for key signaling pathways via gene set enrichment analysis (GSEA).

### 2.5. Cell Viability Assay

U937 cells, NB4 cells, and HL60 cells were purchased from the National Collection of Authenticated Cell Cultures (Shanghai, China). MOLM-13 cells and MV4-11 cells were purchased from BeNa Culture Collection (Xinyang, China). Triptonide (TN) was purchased from Chengdu Must Bio-Technology Co., Ltd. (Chengdu, China), and the purity was determined to be 99.72% (MUST-23122409). TN was dissolved in dimethyl sulfoxide (DMSO) (Solarbio, Beijing, China) as a stock solution of 100 mM, stored at −80 °C, and diluted with cell culture medium before each experiment.

We plated 5 × 10^3^ cells in the logarithmic phase into 96-well plates and treated them with TN at concentrations ranging from 0 to 160 nM for 24, 48, and 72 h at 37 °C. After treatment, CCK8 reagent (MeilunBio, Dalian, China) was dispensed into each well, and the plates were incubated at 37 °C for 2 h. Finally, the cell viability results were analyzed using a microplate reader at 450 nm to quantify absorbance.

### 2.6. Apoptosis Assay

Apoptosis was assessed via the Annexin V-YSFluor™ 647/7-AAD Apoptosis Detection Kit (YEASEN, Shanghai, China). We seeded U937 cells (5 × 10^5^ cells/well) into 6-well plates and treated them with TN at concentrations of 0, 10, 20, and 40 nM for 72 h. Then, we washed the cells twice with PBS and resuspended them in 1× binding buffer. Next, the cells were stained with 5 μL of Annexin V-YSFluor™ 647 and then incubated at room temperature for 5 min in the dark. 7-AAD (10 μL) was subsequently added. Flow cytometry analysis was performed within 30 min using a FACSCalibur instrument (BD Biosciences, San Jose, CA, USA) with FlowJo (version 10.8.1) (FlowJo, Ashland, OR, USA) for data visualization. For MOLM-13 cells, Annexin V-FITC/PI Apoptosis Detection Kit (BD, Franklin Lakes, NJ, USA) was performed simultaneously following the same principle with optimized procedures.

### 2.7. Western Blotting

U937 and MOLM-13 cells (5 × 10^5^ cells/well) were seeded into 6-well plates and treated with TN at concentrations of 0, 10, 20, and 40 nM for 72 h, with or without co-treatment of the AKT agonist SC79 (HY-18749, MCE, Shanghai, China). Subsequently, Western blot analysis was performed to assess protein expression under these conditions.

Total proteins were isolated from U937 and MOLM-13 cells with RIPA lysis buffer (Solarbio, Beijing, China) supplemented with protease inhibitors, quantified via a BCA assay (Beyotime, Shanghai, China), and separated on 10–12% SDS-PAGE gels at 120 V for 90 min. The proteins were subsequently transferred to PVDF membranes (Millipore, Billerica, MA, USA) by electroblotting. The membranes were blocked and subsequently incubated overnight at 4 °C with the following primary antibodies: β-actin (20536-1-AP) and GAPDH (10494-1-AP) were purchase from Proteintech (Rosemont, IL, USA). phospho-AKT (#4060) and Cleaved-caspase 3 (#9664) were obtained from Cell Signaling Technology (Beverly, MA, USA). phospho-PI3K (AF3242) and Goat anti-rabbit lgG (H+L) HRP (S0001) were purchased from Affinity Biosciences (Liyang, China). The membranes were washed and subsequently incubated with the secondary antibody for 2 h. Finally, the protein bands were visualized and quantified via an enhanced chemiluminescence (ECL) system (Bio-Rad, Hercules, CA, USA) and Image Lab (Bio-Rad, Hercules, USA).

### 2.8. Animal Experiments

Female BALB/c nude mice (4–5 weeks, 18–20 g) acquired from Beijing Vital River Laboratory Animal Technology Co., Ltd. (Beijing, China), were housed under SPF conditions. The animal experiments were authorized by the Experimental Animal Ethics Committee of the Institute of Chinese Materia Medica, China Academy of Chinese Medical Sciences (Approval No. 2023B022). U937 cells (5 × 10^6^/200 μL in saline) were subcutaneously injected into the right flank. When the tumors reached approximately 100 mm^3^, the mice were randomized into three groups: Control (CMCNA), TN-L (low dose) (5 mg/kg), and TN-H (high dose) (10 mg/kg). Each group of mice received intragastric administration of TN or equivalent doses of CMCNA once daily for 13 consecutive days. The body weight and tumor volume (length × width^2^)/2 were monitored. After 13 d of orally administered, mice were humanely euthanized via CO_2_ asphyxiation using a controlled gas delivery system, and tumor tissues were collected for subsequent evaluation. The organs (liver, spleen, kidney, heart) were excised for histopathology (4% PFA (Solarbio, Beijing, China)).

### 2.9. Hematoxylin and Eosin (H&E) Staining

Heart, liver, spleen, and kidney tissues were fixed in 4% PFA for 36 h, dehydrated with graded ethanol, embedded in paraffin, and sectioned into 4-μm-thick slices. These sections were subjected to H&E staining to evaluate histopathological alterations and subsequently mounted and observed under a microscope (PerkinElmer, Waltham, MA, USA).

### 2.10. Immunohistochemical Staining

Paraffin-embedded sections were prepared for immunohistochemistry (IHC) analysis. The tissue sections were deparaffinized, hydrated, and permeabilized with 0.5% Triton X-100 in 1× PBS for 10 min, followed by blocking with 1% BSA. Primary antibodies (Ki67, ab16667, Abcam, Waltham, MA, USA) were incubated overnight at 4 °C. Sections were washed three times with PBS and incubated with HRP-conjugated secondary antibody. DAB chromogen was applied for color development, and hematoxylin was used for counterstaining. Slides were imaged under a microscope and analyzed for Ki67-positive cells using the Image-Pro Plus software (v.6.0) (Media Cybernetics, Rockville, MD, USA).

### 2.11. Statistical Analysis

All the statistical analyses were conducted via GraphPad Prism 8.0 (GraphPad Software, San Diego, CA, USA). One-way ANOVA was used to compare three or more groups, while t tests were employed for comparisons between two groups. All the data are presented as the means ± SEMs, with *p* < 0.05 considered statistically significant.

## 3. Results

### 3.1. Network Pharmacology Analysis of TN in AML Treatment

**Figure 2:**
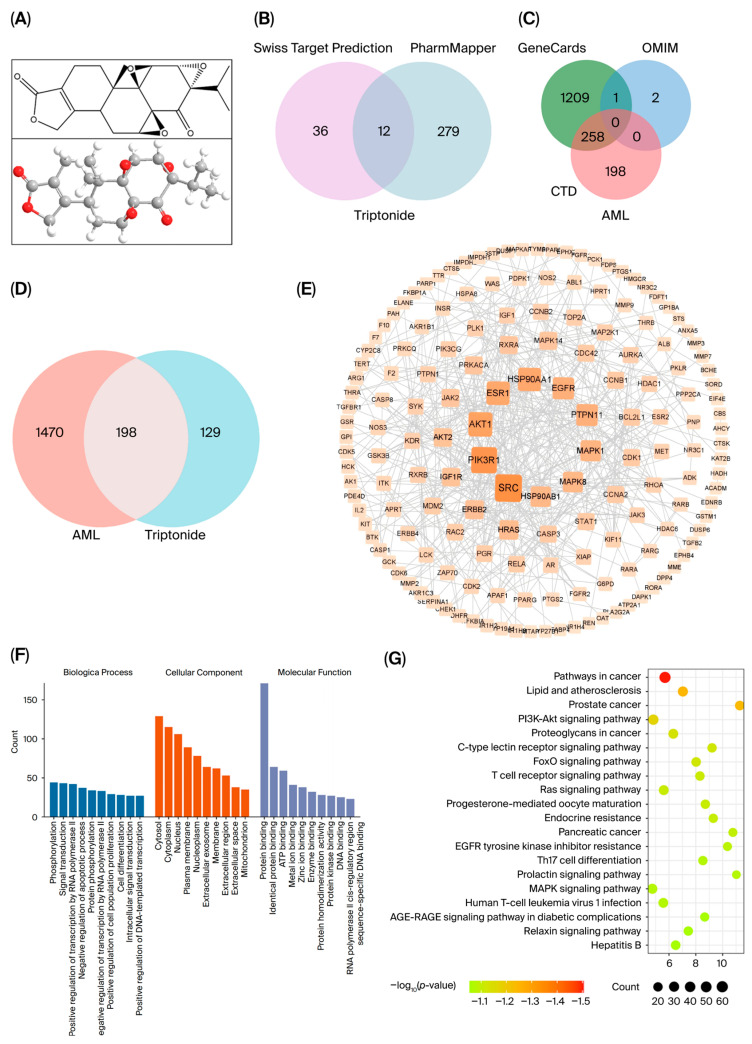
Integrated network pharmacology analysis of TN in AML therapy. (**A**) Structural formula of TN from the PubChem database. (**B**) Integrated TN targets derived from Swiss Target Prediction and PharmMapper. (**C**) Integrated AML targets derived from GeneCards, OMIM, and CTD database. (**D**) Venn diagram illustrating the overlap of candidate targets for TN and AML. (**E**) PPI network of the 198 overlapping genes constructed using the STRING database and Cytoscape software. (**F**) GO enrichment analysis of the 198 overlapping genes, illustrating BP, MF, and CC involved. Blue represents the top 10 BP, Orange indicates the top 10 CC, and Purple denotes the top 10 MF. (**G**) KEGG pathway enrichment analysis of the 198 overlapping targets, highlighting relevant signaling pathways and mechanisms.

The 2D and 3D structures of the TNs are depicted in Figure 2A. Through a search in the Swiss Target Prediction and PharmMapper target modules, we identified 327 putative targets linked to TNs, as well as their corresponding gene symbols (Figure 2B, Table S1). After the integration of AML-associated targets from GeneCards, CTD, and OMIM and the removal of duplicates, 1668 disease targets were identified (Figure 2C, Table S2). The 198 overlapping targets were recognized as core targets shared with TN targets and AML targets in a Venn diagram (Figure 2D, Table S3).

To determine targets with direct or indirect interactions involved in the treatment of AML with TN, a PPI network was constructed from 198 common targets using STRING and Cytoscape (Figure 2E). The PPI network comprises 164 nodes and 453 edges, with node color intensity exhibiting a gradient from light to dark to represent increasing degree centrality values across the network.

For the functional analysis of TN in AML, 198 targets were subjected to GO and KEGG analyses. The results are visualized in a bar chart and a scatter diagram. The GO results revealed significant functional enrichment across three ontological domains: BP, CC, and MF (Tables S4–S6). Notably, the top 10 enriched terms demonstrated pronounced biological relevance, with “phosphorylation” and “signal transduction” emerging as the most significantly enriched terms, whereas “protein binding” dominated the MF category (Figure 2F). KEGG analysis revealed 153 significantly enriched pathways (*p* < 0.05, FDR < 0.05). The top 20 pathways, including ‘Pathways in cancer’, ‘Lipid and atherosclerosis’, ‘Prostate cancer’, and ‘PI3K/AKT signaling pathway’, were visualized (Figure 2G, Table S7).

**Figure 3:**
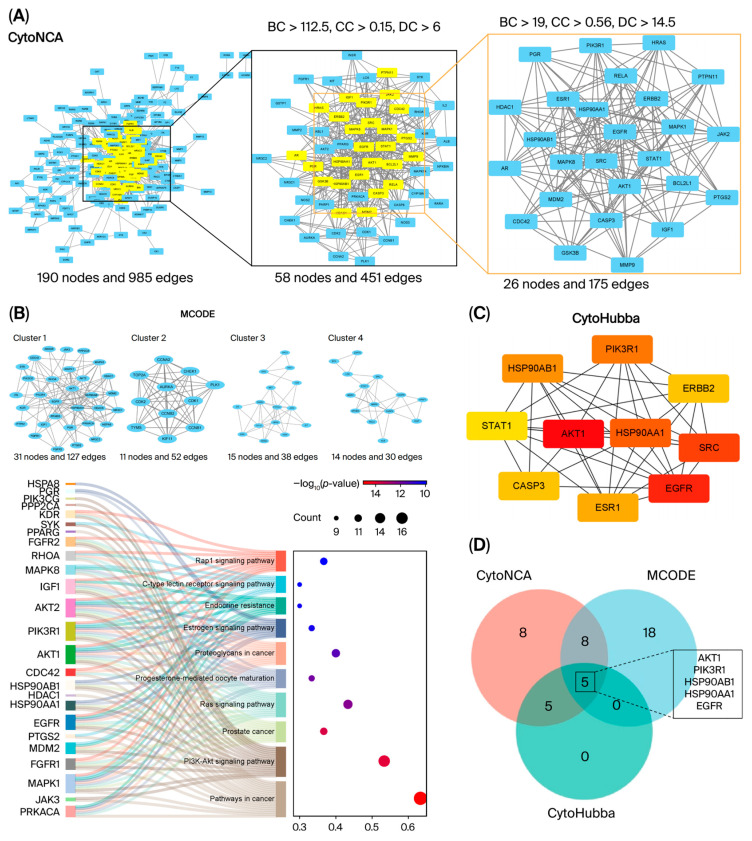
Identification of core genes. The hub genes screening using three topological analysis algorithms: (**A**) CytoNCA algorithm calculates node centrality metrics (degree, closeness, betweenness) to quantify network influence. (**B**) MCODE plugin detects densely connected functional clusters through density-based modularization. KEGG enrichment analysis of Cluster 1. (**C**) CytoHubba employs a degree algorithm to prioritize interaction-dense nodes. The darker the color of the target, the greater the number of interactions. (**D**) Venn diagram of key targets identified by different algorithms showing intersections.

The topological characteristics of the network nodes were systematically evaluated through CytoNCA, which incorporates three critical centrality metrics: betweenness centrality (BC), closeness centrality (CC), and degree centrality (DC). The initial screening employed median threshold criteria of 190 genes (BC > 112.5, CC > 0.15, DC > 6), which identified 58 candidate genes. Subsequent, 58 candidate genes were employed to calculate the topological parameters again by using CytoNCA, secondary screening applied stricter median thresholds (BC > 19, CC > 0.56, DC > 14.5) to refine the core targets, ultimately isolating 26 pivotal interacting genes demonstrating robust topological prominence (Figure 3A). The detailed information of the 26 targets, ranked by degree, is shown in Tables S8–S10.

To further explore the functional importance of these targets, we utilized the MCODE plugin to conduct cluster analysis. The targets were categorized into 11 distinct groups, with four main clusters shown. As depicted in Figure 3B, Cluster 1 has 31 nodes and 127 edges, and Cluster 2 has 11 nodes and 52 edges (Table S11). We also performed KEGG analysis of Cluster 1. As shown in Figure 3B, the targets of Cluster 1 were enriched in “Pathways in cancer” and “PI3K/AKT signaling pathway”.

Moreover, the top 10 interacting genes were identified via maximal clique centrality (MCC) via CytoHubba analysis (Figure 3C, Table S12). Darker colors represent higher degree values.

The intersection analysis of the three methods revealed 5 core genes: AKT1, PIK3R1, HSP90AA1, HSP90AB1, and EGFR (Figure 3D, Table 2).

### 3.2. Molecular Docking of TN and the Hub Proteins

**Figure 4:**
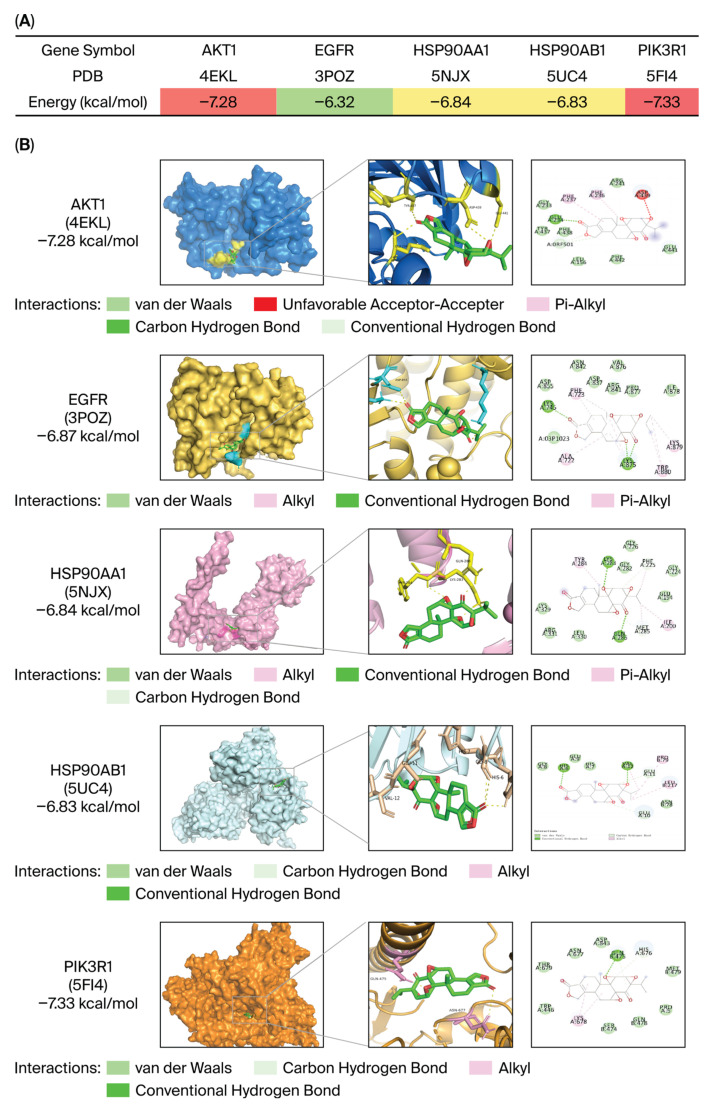
Molecular docking of TN with core genes. (**A**) Binding affinity table of TN with AKT1, EGFR, HSP90AA1, HSP90AB1, and PIK3R1, where darker colors indicate stronger binding energy. (**B**) Binding sites of TN with AKT1, EGFR, HSP90AA1, HSP90AB1, and PIK3R1, with dashed lines indicating hydrogen bonds.

Molecular docking analysis disclosed that TN strongly interacted with five hub proteins (AKT1, EGFR, HSP90AA1, HSP90AB1, and PIK3R1) identified through network pharmacology, exhibiting binding energies between −7.33 and −6.32 kcal/mol (Figure 4A,B, Table S13). AKT1 (PDB:4EKL) and PIK3R1 (PDB:5FI4) demonstrated the highest affinities (−7.28 and −7.33 kcal/mol, respectively), suggesting their critical roles in the pharmacological effects of TN. The binding conformation of AKT1 involves hydrogen bonds with GLU234, whereas that of PIK3R1 involves stable interactions through hydrogen bonds with GLN457. These results indicate that TN may exhibit its biological activity by modulating the PI3K/AKT signaling pathway, providing a mechanistic basis for its antitumor potential.

### 3.3. DEGs Screening and PI3K/AKT Pathway Enrichment Analysis via the GEPIA2 Database

**Figure 5:**
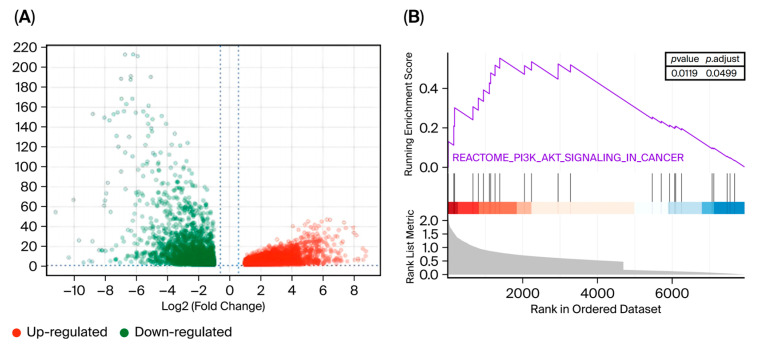
Integrative analysis of AML DEGs and GSEA analysis from the GEPIA 2 clinical database. (**A**) Volcano plot of DEGs between Normal samples and AML samples from the GEPIA 2 database. (**B**) GESA enrichment analysis of PI3K/AKT signaling pathway in AML DEGs.

DEGs between normal samples and AML samples were identified via GEPIA 2. On the basis of the screening criteria of *p* < 0.01 and |Log2FC| > 1, we identified 2706 upregulated and 3254 downregulated genes (Figure 5A). GSEA of the DEGs revealed significant enrichment and upregulation of PI3K/AKT signaling in AML samples (*p* = 0.0119, NES = 1.4) (*p* < 0.05) (Figure 5B).

### 3.4. Molecular Dynamics Simulation for TN and Core Protein

**Figure 6:**
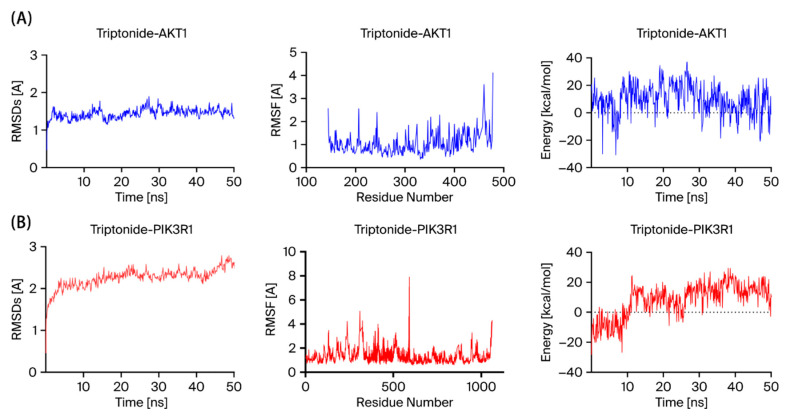
Molecular dynamics simulations of TN with AKT1 and PIK3R1. The RMSD, RMSF, and Energy changes of the complex of triptonide-AKT1 (**A**) and triptonide-PI3KR1 (**B**) in the process.

MD simulations of the docked complex were utilized to confirm the results of the docking study, and the dynamic behavior of the complex was examined to assess its stability. The RMSD curves stayed relatively stable over 50 ns of MD simulations, indicating the stability of TN docking with the AKT1 and PIK3R1 complex conformations (Figure 6A,B).

### 3.5. Effects of TN on U937 Cell Proliferation

**Figure 7:**
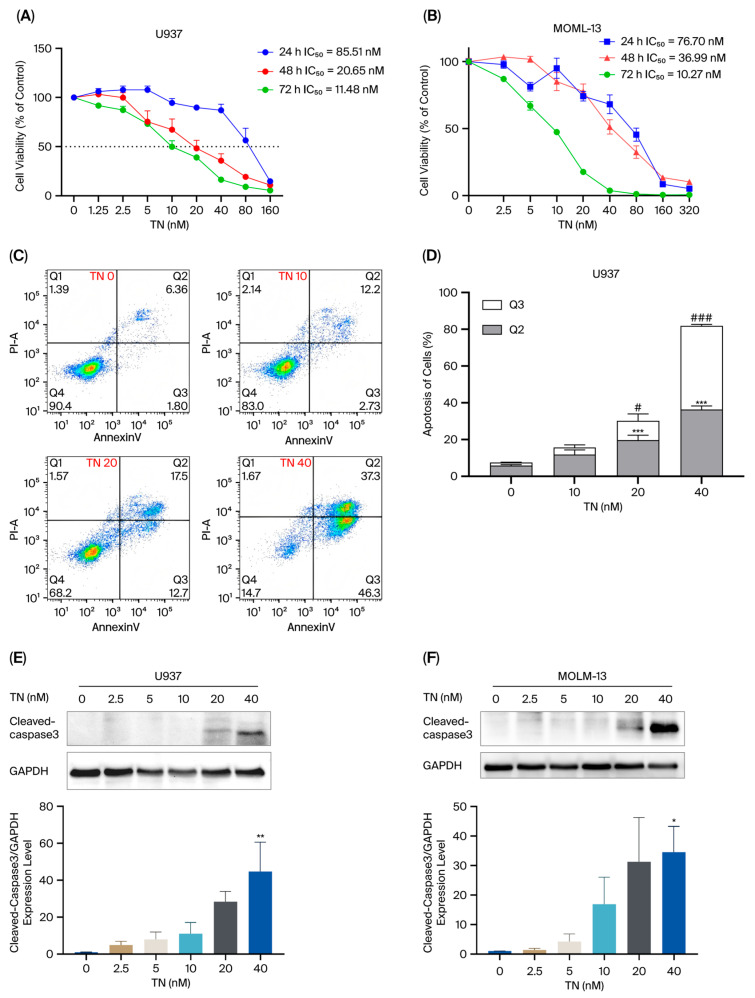
TN inhibits the proliferation and induces apoptosis in U937 and MOLM-13 cells. (**A**,**B**) CCK-8 assays were used to determine the IC_50_ values of TN in U937 and MOLM-13 cells (n = 6). (**C**,**D**) Apoptosis analysis was determined by flow cytometry in U937 cells (n = 3). Compared with the Control group (Q2), *** *p* < 0.001; Compared with the Control group (Q3), ^#^ *p* < 0.05, ^###^ *p* < 0.001. (**E**,**F**) Western blot analysis of Cleaved-caspase 3 protein in TN-treated U937 and MOLM-13 cells (n = 3). Compared with the Control group. * *p* < 0.05; ** *p* < 0.05. Data are presented as mean ± SEM.

To investigate the effects of TN on AML cell lines (U937, MOLM-13, NB4, MV4-11, HL60), cells were exposed to TN at various concentrations for 24 h, 48 h, and 72 h. As shown, the results demonstrated that TN inhibited AML cell proliferation in a concentration- and time-dependent manner (Figure 7A,B, Figure S1). Among these, U937 and MOLM-13 cells exhibited particularly significant inhibitory responses. Specifically, the IC_50_ values of TN in U937 cells were 85.51 nM, 20.71 nM, and 11.52 nM at 24 h, 48 h, and 72 h, respectively (Figure 7A). For MOLM-13 cells, the IC_50_ values were 78.7 nM, 36.99 nM, and 10.27 nM at the respective time points (Figure 7B). Given that the two cell lines are genetically distinct, they were selected for subsequent in vitro experiments.

Flow cytometry analysis with Annexin V-YSFluor™ 647/7-AAD double staining revealed a concentration-dependent effect of TN on U937 and MOLM-13 cell apoptosis (Figure 7C,D, Figure S2). In U937 cells, as the TN concentration increased, the proportion of viable cells (Q4) decreased (from 91.17% at 0 nM to 16.33% at 40 nM), while the early (Q2) and late apoptotic populations increased (from 5.89% to 36.3% and from 1.60% to 45.57%, respectively) (Figure 7C,D). A similar trend was observed in MOLM-13 cells, with viable cells (Q4) decreasing from 95.30% to 21.97%, early apoptotic cells (Q2) increasing from 1.83% to 28.07%, and late apoptotic cells (Q3) from 0.26% to 46.27% at the same concentration (Figure S2). Consequently, the total apoptotic cell population (Q2 + Q3) was significantly elevated in both cell lines after TN treatment (*p* < 0.001). Meanwhile, the apoptosis-related protein Cleaved-caspase 3 was markedly upregulated in U937 and MOLM-13 cells treated with 20 and 40 nM TN (*p* < 0.05) (Figure 7E,F). These results indicated that TN induces apoptosis in AML cells.

**Figure 8:**
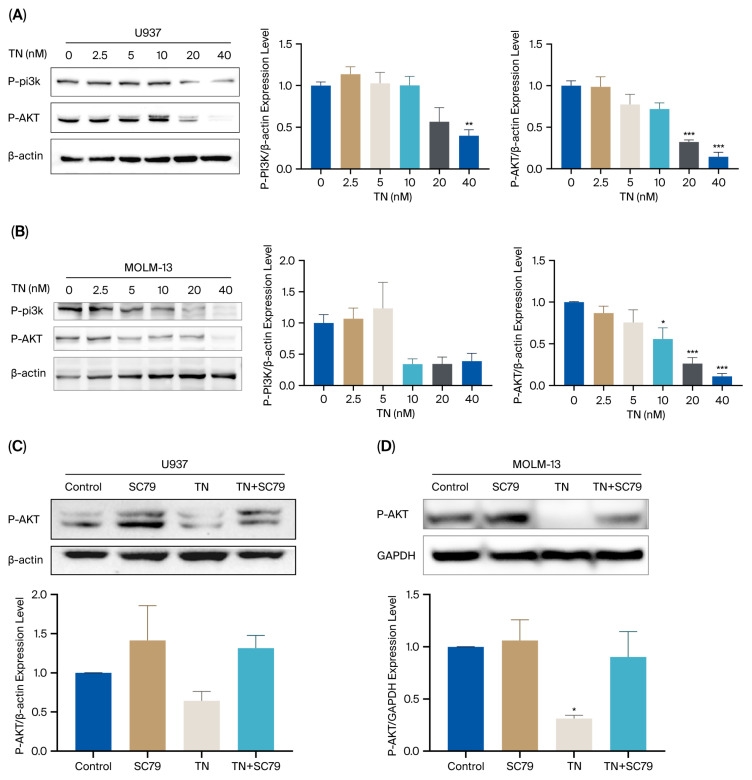
Effects of TN on U937 and MOLM-13 cells in vitro. (**A**,**B**) Western blot analysis of P-PI3K and P-AKT protein in TN-treated U937 and MOLM-13 cells (n = 3). (**C**,**D**) Western blot analysis of P-AKT in TN-treated U937 and MOLM-13 cells treated with SC79 (AKT activator) (n = 3). Data are presented as mean ± SEM. Compared with the Control group. * *p* <0.05, ** *p* <0.01, *** *p* <0.001.

Subsequently, we validated the protein levels of core targets identified through network pharmacology and molecular docking. Western blot analysis revealed that TN (40 nM) markedly downregulated P-AKT and P-PI3K protein levels in U937 and MOLM-13 cells (Figure 8A,B), suggesting TN-mediated inhibition of the PI3K/AKT pathway. To further confirm this mechanism, rescue experiments were performed using SC79, an AKT activator. As shown in Figure 7E, SC79 treatment reversed the TN-induced suppression of P-AKT levels.

### 3.6. TN Effectively Inhibits Tumor Formation in U937 Cells in Vivo

**Figure 9:**
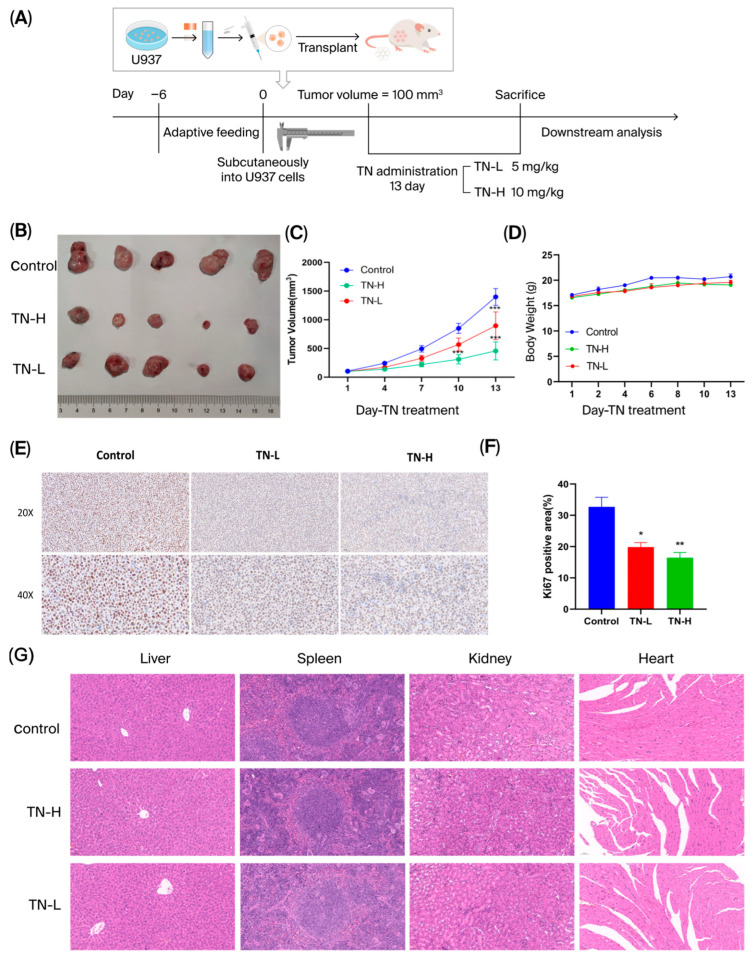
The influence of TN on the xenograft model in nude mice. (**A**) Outline of animal experiments. (**B**) Tumor masses are derived from three groups. (**C**,**D**) Changes in tumor volume and body weight after treatment with vehicle or TN (n = 6). (**E**,**F**) Immunohistochemical analysis of Ki67 in tumor sections of the three groups (n = 3). (**G**) HE staining of liver, spleen, kidney, and heart tissues in three groups. Compared with the Control group, * *p* < 0.05, ** *p* < 0.01.

We evaluated the therapeutic efficacy of TN against AML in vivo via a xenograft model established, as illustrated in Figure 9A. When the tumor volume reached about 100 mm^3^ in total volume, mice were administrated with either TN daily or the CMCNA for 13 days. Tumor volumes were monitored every 3 days. Tumor growth curve results confirmed that TN administration potently inhibited U937 xenograft tumor growth in nude mice (*p* < 0.01) (Figure 9B,C). Quantitative IHC analysis using ImageJ software (version 1.8.0_345) (National Institutes of Health, Bethesda, USA) revealed a significant reduction in Ki67-positive proliferative areas in the TN-H group versus the model group (*p* < 0.05) (Figure 9E,F).

To assess the safety of TN in vivo, major organs (heart, liver, spleen, and kidney) were analyzed via HE staining to detect pathological changes. No significant toxicity was observed in these organs following TN administration (Figure 9G). These results indicated that TN could inhibit U937 xenograft tumors growth in vivo without obvious side effects.

## 4. Discussion

AML is a highly invasive hematologic malignancy and is marked by poor survival outcomes due to its invasive nature and treatment resistance. Current first-line therapies, such as the “7 + 3” regimen, often fail in elderly patients because of severe myelosuppression and treatment-related toxicity [27,28]. In recent years, the multitarget and low-toxicity characteristics of traditional Chinese medicine have emerged as promising directions for anticancer therapy [29,30]. Among these compounds, TN, a diterpenoid from *Tripterygium wilfordii*, has attracted significant attention as a promising anticancer agent because of its broad antitumor activities and wider therapeutic window. TN exhibits potent antitumor effects through multitarget mechanisms, including the induction of apoptosis via p38 MAPK/p53 activation in AML [15] and the suppression of Wnt/β-catenin signaling in colorectal cancer [31]. It also demonstrates broad-spectrum efficacy against solid tumors (e.g., gastric, lung, and prostate cancers) by targeting the Notch1/NF-κB pathways to inhibit metastasis and mTOR signaling to combat drug resistance in prostate cancer [22,32].

In this study, we investigated the mechanism through which TN affects AML. First, we evaluated the anti-AML activity of TN using the CCK8 assay. Given the genetic heterogeneity typical of AML, we selected a panel of cell lines, including U937, MOLM-13, NB4, MV4-11, and HL60. TN treatment strongly inhibited proliferation across all tested lines, with IC_50_ values of 11.48 nM in U937, 10.27 nM in MOLM-13, 21.67 nM in NB4, 13.69 nM in MV4-11, and 42.49 nM in HL60 at 72 h. The consistent inhibitory effect across genetically diverse backgrounds suggests that TN may function as a pan-AML therapeutic agent. To further elucidate its mechanism of action, we adopted an integrated approach combining network pharmacology, molecular docking, MD simulation, and experimental validation.

The network pharmacology was employed to construct a compound-target-disease interaction network. This approach involves the integration of multiple databases to avoid omissions in single database sources and the application of diverse network analysis approaches to identify core targets. MCODE analysis utilized clusters with consistent functional annotations to choose seed genes, whereas CytoNCA conducted network centrality analysis via topological metrics (BC, CC, DC) to assess node importance and identify critical regulators. The CytoHubba plug-in, further identified through MCC analysis, prioritizes core targets on the basis of their structural influence within the network.

In this study, 327 potential targets associated with TN were acquired from the Swiss Target Prediction and PharmMapper databases. A total of 1668 disease targets linked to AML were acquired from OMIM, GeneCards, and CTD. A total of 198 co-expressed targets of TN with AML were identified via a Venn diagram. GO analysis revealed the biological relevance of TN with AML, whereas KEGG pathway analysis revealed the following key signaling pathways: pathways in cancer, lipid and atherosclerosis, prostate cancer, and PI3K/AKT. Finally, we integrated five core targets, AKT1, EGFR, HSP90AA1, HSP90AB1, and PIK3R1, via Cytoscape plugin analysis (CytoNCA, MCODE, and CytoHubba).

Molecular docking was carried out to clarify the molecular interactions of the complexes. Molecular docking analysis revealed stable interactions between TN and AKT1 (binding energy: −7.28 kcal/mol) and PIK3R1 (binding energy: −7.33 kcal/mol), both of which exceeded the −7 kcal/mol threshold for significant drug-target affinity. Hydrogen bonds are highly specific interactions between a receptor and a ligand, critical in stabilizing protein-ligand complexes. By forming a hydrogen bond with the GLU234 residue in AKT1, TN enhances its affinity, blocks substrate binding to AKT1, and thereby inhibits AKT1’s kinase activity. Additionally, TN establishes a hydrogen bond with the GLN457 residue of PIK3R1, a critical residue located within the PI3K binding domain. TN inhibits PI3K activity by binding to the GLN457 residue.

MD simulations support the molecular docking results of this study to demonstrate the stability of TN in the framework of MD simulations. RMSD value describes the conformational shift in a macromolecule that changes into a receptor after coming into contact with a specific ligand. Lower RMSD values suggest greater conformational stability. In this study, TN showed a favorable interaction between considerable conformational shift and bond relaxation, staying below 3.0 Å, indicating a particularly stable interaction structure. MD simulation techniques confirmed the reliability of the docking of TN with the AKT1 and PIK3R1 conformations.

AKT1, known as protein kinase B, serves a vital function in tumorigenesis by modulating cancer cell proliferation, survival, and apoptosis. Hyperactivation and mutation of AKT1 contribute to the pathogenesis of various cancers, including lung, ovarian, pancreatic, and leukemia [33].

PIK3R1, encoding the PI3K regulatory subunit p85α, sustains PI3K activity to facilitate LSC self-renewal. Its mutations have been identified in leukemia [34] and other solid tumors, often leading to constitutive PI3K/AKT pathway overactivation, which is linked to cancer progression [35].

MCODE plugin analysis revealed that the core target cluster (Cluster 1) was significantly enriched in the PI3K/AKT signaling pathway, with its enrichment significance ranking second only to “Pathways in Cancer”. GEPIA 2 database analysis revealed elevated mRNA levels of PIK3R1 and AKT1 in AML patients. Furthermore, GSEA validated the significant activation of this pathway in AML (normalized enrichment score > 1, *p* < 0.05). These findings suggest that the PI3K/AKT signaling pathway is critical for the development of leukemia, which is consistent with reports that AKT1 overactivation promotes tumorigenesis and that PIK3R1 mutations are significantly linked to poor prognosis in hematologic malignancies [33,36,37]. MD simulations revealed the stability of TN docking to the AKT1 and PIK3R1 complex conformations. Furthermore, Western blot analyses proved that TN downregulated the expression of AKT and PI3K in AML cells, suggesting that TN may exert antileukemic effects through inhibition of this pathway. Additionally, we employed an AKT activator SC79 to verify the molecular mechanism. SC79 enhances AKT phosphorylation through direct binding to its PH domain, inducing conformational changes and activating the kinase. Experimental validation using combined treatment with TN and SC79 demonstrated significant the AKT activation, further confirmed that the therapeutic action of TN is mediated through modulation of the AKT. These findings provide a theoretical basis for targeting this pathway in leukemia therapy.

The PI3K/AKT pathway is aberrantly activated in diverse cancers and serves a key function in proliferation and survival [38,39]. Dysregulation of this pathway—characterized by hyperactivation of PI3K and gain-of-function of AKT—is a notorious drivers of disease progression in cancer [40,41]. The existing literature has reported that TN inhibit HCC proliferation and inducing apoptosis by repressing PI3K/AKT signaling [42]. In this study, we extend these findings to AML and demonstrated that TN significantly suppressed AML cell proliferation by triggering apoptosis in a dose-dependent manner. This apoptotic effect aligns with the mechanisms observed in other antitumor agents targeting the PI3K/AKT axis [39].

To further investigate the efficacy of TN on AML, we established a xenograft tumor model with U937 cells in nude mice. The results demonstrated that TN (5–10 mg/kg) reduced the tumor volume and Ki67-positive areas, and did not cause pathological changes in organ, indicating the effectiveness and safety of TN in treating AML. Of note, this study has a limitation in that it lacks toxicological data on TN in mice. The toxicology of TN-mediated therapy against AML remains to be systemically investigated.

Our study introduces novel insights and, for the first time, predicts and identifies the potential binding relationship between TN with AKT1 and PIK3R1 through network pharmacology-based analysis, which was further validated by molecular docking and molecular dynamics simulations, establishing a PI3K-AKT axis regulatory mechanism in AML. Additionally, a TN-AML multi-target regulatory network was constructed, elucidating the synergistic multi-target regulatory effects of TN and overcoming the limitations of traditional single-target studies, thereby providing a paradigm for elucidating the multi-target mechanisms of natural products. Currently, no PI3K inhibitors or AKT inhibitors targeting AML have been approved as first-line treatment drugs [43]. In this study, the natural product TN was demonstrated to be able to inhibit this pathway, and it may become a potential drug for PI3K-AKT inhibitors. At the same time, the multi-target characteristic of TN may reduce the risk of drug resistance, and TN has shown low toxicity, positioning it as a safer candidate for AML therapy.

Notably, there were certain limitations in this study. In vivo experiments, the sample size (n = 6 per group) may limit the statistical power. While the current sample size provides preliminary evidence for therapeutic efficacy, we should employ a rigorous experimental design to enhance data reliability. Future studies with larger cohorts and power analyses are warranted to validate these findings. Furthermore, a critical limitation of the current study is the use of subcutaneous xenograft models, which may not fully recapitulate the bone marrow microenvironment essential for AML pathogenesis and drug response. Our subcutaneous model, while valuable for initial efficacy assessment, lacks this complex microenvironmental context, potentially limiting the translational relevance of our findings. To address this, we acknowledge the need to validate our findings using orthotopic models, such as intravenous transplantation of AML cell lines into the mice, which better mimic hematopoietic niche interactions.

Future research should focus on core areas to advance TN as a novel AML therapy. Combination strategies with hypomethylating agents (e.g., azacitidine) or BCL-2 inhibitors (e.g., venetoclax) should be evaluated to overcome chemoresistance, leveraging the synergistic PI3K/AKT inhibitory effects observed in preclinical models. In addition, myeloid differentiation plays a pivotal role in AML treatment [44]. Against this backdrop, whether TN promoted myeloid differentiation by regulating differentiation-related pathways remains an open scientific question that will constitute a key focus of our subsequent investigations. Nevertheless, our findings establish a foundational framework for developing TN as a promising candidate for AML treatment.

## 5. Conclusions

In conclusion, we integrated network pharmacology, bioinformatics, and experimental validation (in vivo/in vitro) to investigate the potential therapeutic targets of TN in AML. Through network pharmacology analysis, the core genes were identified as potential therapeutic targets, with significant enrichment observed for AKT1, EGFR, HSP90AA1, HSP90AB1, and PIK3R1. By integrating the binding energy of TN with clinical data, PIK3R1 and AKT1 were particularly highlighted as key targets of TN. Moreover, our experimental results validated these bioinformatic analyses, demonstrating that TN significantly downregulated the protein expression of AKT and PI3K in AML cells. Furthermore, TN effectively restrained cell proliferation in vitro and reduced tumor growth in vivo. In brief, TN may modulate the PI3K/AKT signaling pathway to exert a powerful anti-AML effect. These results not only offer novel insights into the anti-AML mechanism of TN but also lay a basis for developing targeted therapies while offering new research ideas and strategies for targeted investigations.

## Figures and Tables

**Table 1:** The website software used in the study.

No.	Software/Web Tool /Database	URL
1	CTD database	https://ctdbase.org/ (accessed on 15 January 2024)
2	GeneCards database	https://www.genecards.org/ (accessed on 15 January 2024)
3	OMIM database	https://omim.org/ (accessed on 15 January 2024)
4	PharmMapper	https://www.lilab-ecust.cn/pharmmapper/ (accessed on 30 October 2024)
5	Swiss Target Prediction	http://www.swisstargetprediction.ch/ (accessed on 12 January 2024)
6	STRING database	https://cn.string-db.org/ (accessed on 30 October 2024)
7	DAVID database	https://davidbioinformatics.nih.gov/ (accessed on 30 October 2024)
8	PDB database	https://www.rcsb.org/ (accessed on 1 August 2025)
9	PubChem database	https://pubchem.ncbi.nlm.nih.gov/ (accessed on 3 April 2025)
10	AutoDock Vina_v1.1.2	https://vina.scripps.edu/ (accessed on 7 August 2025)
11	Cytoscape_v3.8.0	https://cytoscape.org/ (accessed on 6 August 2025)
12	Cytoscape NCA	https://apps.cytoscape.org/apps/cytonca (accessed on 5 August 2025)
13	Cytoscape Hubba	https://apps.cytoscape.org/apps/cytohubba (accessed on 6 August 2025)
14	Cytoscape MCODE	https://apps.cytoscape.org/apps/mcode (accessed on 6 August 2025)
15	Open Babel software	https://openbabel.org/index.html (accessed on 6 August 2025)
16	Pymol_v3.1.4	https://www.pymol.org/ (accessed on 6 August 2025)
17	Uniprot database	https://www.uniprot.org/ (accessed on 6 August 2025)
18	Microbio informatics platform	https://www.bioinformatics.com.cn/ (accessed on 1 August 2025)
19	GEPIA2 database	http://gepia2.cancer-pku.cn/#index (accessed on 8 August 2025)
20	PYMOL 4.6.0	https://pymol.org/ (accessed on 1 August 2025)
21	BIOVIA Discovery Studio	https://www.3ds.com/products/biovia/discovery-studio (accessed on 7 August 2025)

**Table 2:** The key targets were determined through the intersection of three plugins: CytoNCA, MCODE, and CytoHubba.

No.	Uniprot ID	Gene Symbol	Protein Name
1	P31749	AKT1	RAC-alpha serine/threonine-protein kinase
2	P00533	EGFR	Epidermal growth factor receptor
3	P07900	HSP90AA1	Heat shock protein HSP 90-alpha
4	P08238	HSP90AB1	Heat shock protein HSP 90-beta
5	P27986	PIK3R1	Phosphatidylinositol 3-kinase regulatory subunit alpha

## Data Availability

The original contributions presented in this study are included in the article/Supplementary Material. Further inquiries can be directed to the corresponding author(s).

## Supplementary Materials

The following supporting information can be downloaded at: https://www.mdpi.com/article/10.3390/cimb48030239/s1.

## Abbreviations

AML	Acute myeloid leukemia
BP	Biological Process
CC	Cellular Component
DEGs	Differentially expressed genes
GO	Gene Ontology
GSEA	Gene set enrichment analysis
HSCT	Hematopoietic stem cell transplantation
KEGG	Kyoto Encyclopedia of Genes and Genomes
LSCs	Leukemia stem cells
MCC	Maximal clique centrality
MD	Molecular dynamics
MF	Molecular Function
R/R	Relapsed/refractory
RMSDs	Root mean square deviations
RMSFs	Root mean square fluctuations
TCM	Traditional Chinese medicine
TN	Triptonide
