# Supplementary material for: Triptonide Suppresses AML via PI3K/AKT Signaling: A Network Pharmacology Approach Validated by Molecular Docking and Experimental Studies
Source: Curr Issues Mol Biol. 2026 Feb 24;48(3):239. doi: 10.3390/cimb48030239 (PMC13025876; doi:10.3390/cimb48030239)
Supplement: Supplementary file 1 [file cimb-48-00239-s001.zip › cimb-4096056-supplementary/Supplementary material.pdf]

Figure S1

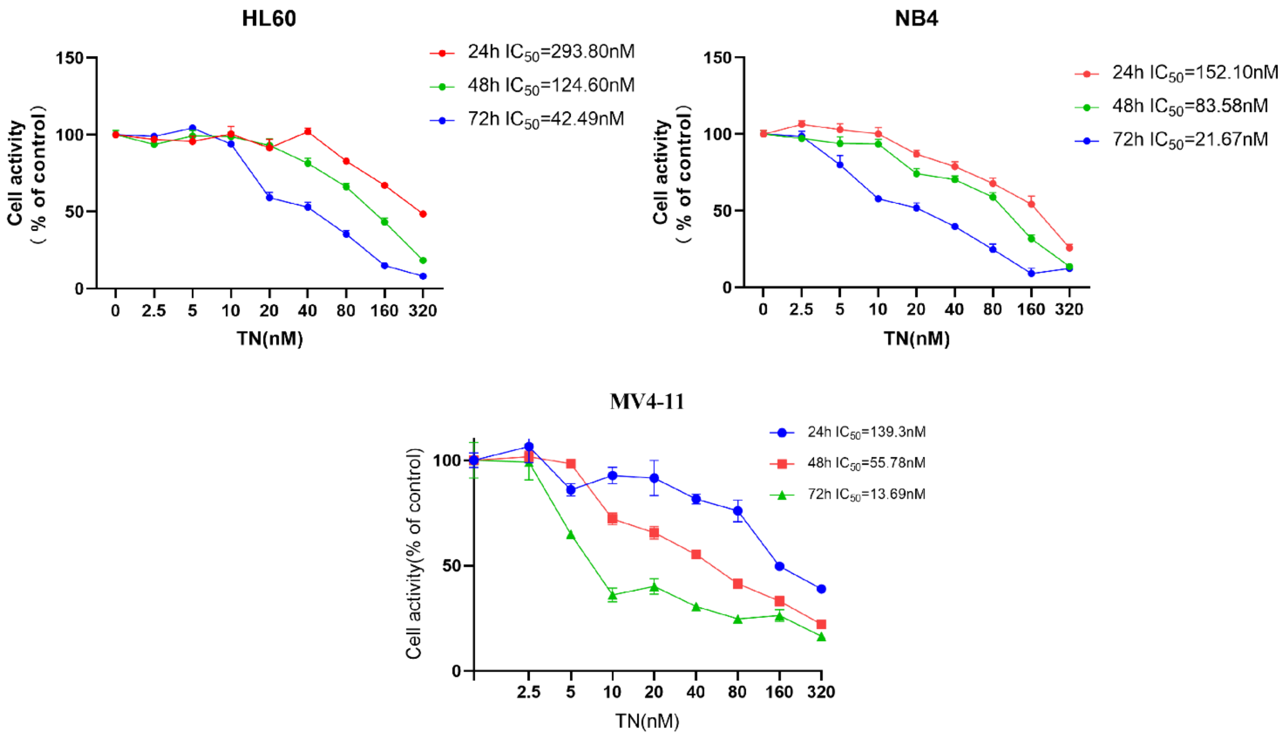

Figure S1. CCK-8 assays were used to determine the IC50 values of TN in HL60, NB4, and MV4-11 cells.

Figure S2

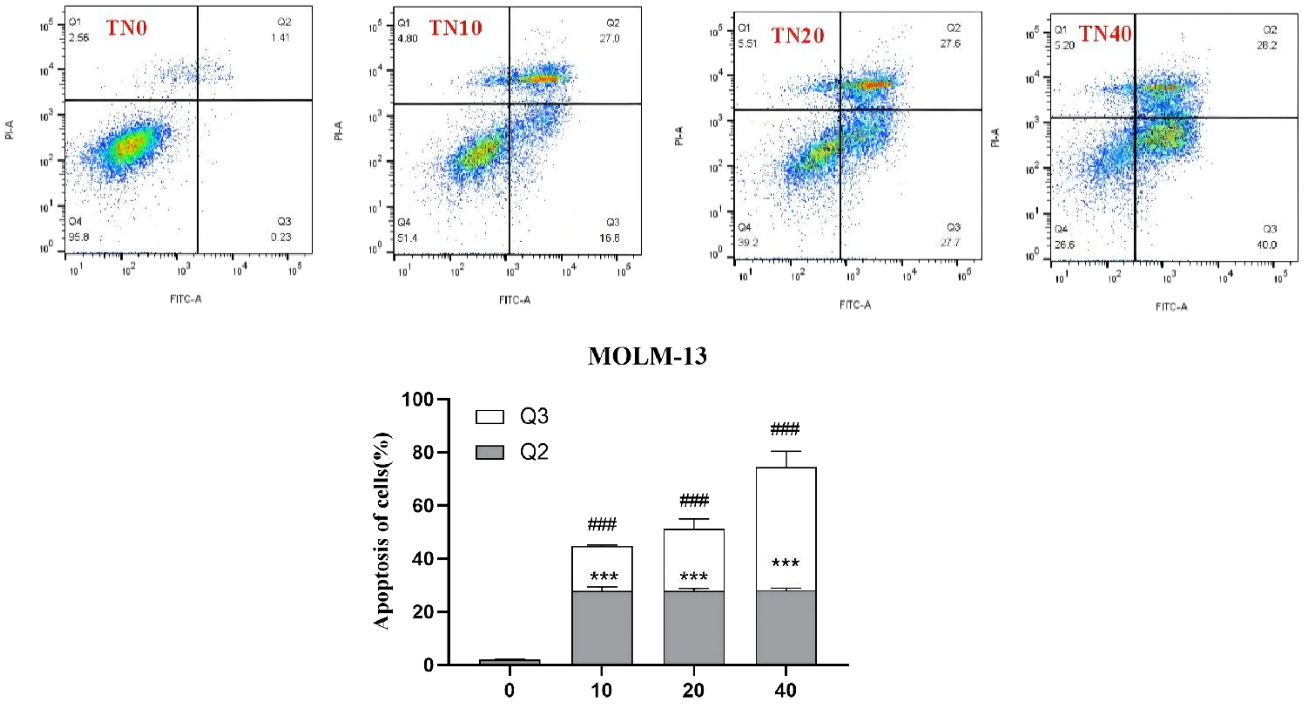

Figure S2. Apoptosis analysis were determined by flow cytometry (n=3) in MOLM-13 cells. Compared with the Control group (Q2), $^{**}P<0.01$, $^{***}P<0.00$timetim1； Compare with the Control group (Q3), $^{\#}P<0.05$, $^{\#\#\#}P<0.001$.

Figure S3 Multi-Database Validation Summary

Target source	Database name	URL	Identified target	Total number of merged targets (after deduplication)	Number of intersecting targets	Overlap(%)
"Triptonide" target	Swiss Target Prediction	http://www.swisstargetprediction.ch/	48	327	198	64.58%
	Pharmmapper	https://www.lilab-ecust.cn/pharmmapper/	291			60.82%
"Acute myeloid leukemia" target	GeneCards	https://www.genecards.org/	1468	1668		12.94%
	CTD	https://ctdbase.org/	456			14.47%
	OMIM	https://omim.org/	3			0.00%
